# Unintended pregnancy and subsequent postpartum long-acting reversible contraceptive use in Zimbabwe

**DOI:** 10.1186/s12905-018-0668-z

**Published:** 2018-11-26

**Authors:** Nerissa Nance, Lauren Ralph, Nancy Padian, Frances Cowan, Raluca Buzdugan, Angela Mushavi, Agnes Mahomva, Sandra I McCoy

**Affiliations:** 1 University of California, Berkeley School of Public Health, Berkeley, USA; 2 Advancing New Standards in Reproductive Health (ANSIRH), University of California, San Francisco, USA; 3 Liverpool School of Tropical Medicine, Pembroke Pl, Liverpool, UK; 4 Centre for Sexual Health and HIV AIDS Research Zimbabwe (CeSHHAR), Harare, Zimbabwe; 5 Ministry of Health and Child Care, Harare, Zimbabwe; 6 Elizabeth Glazier Pediatric AIDS Foundation, Washington, D.C., USA

## Abstract

**Background:**

The postpartum period is an opportune time for contraception adoption, as women have extended interaction with the reproductive healthcare system and therefore more opportunity to learn about and adopt contraceptive methods. This may be especially true for women who experience unintended pregnancy, a key target population for contraceptive programs and programs to eliminate mother-to-child HIV transmission. Among women in Zimbabwe surveyed in 2014, we examined the relationship between pregnancy intention associated with a woman’s most recent pregnancy, and her subsequent postpartum contraceptive use.

**Methods:**

In our analysis we utilized a dataset from a random selection of catchment areas in Zimbabwe to examine the association between pregnancy intention of most recent pregnancy and subsequent postpartum contraceptive use using multinomial logistic regression models. We also explored whether this association differed by women’s HIV status. Finally, we examined the association between pregnancy intention and changes in contraception from the pre- to postpartum periods.

**Results:**

Findings suggest that women who reported that their pregnancy was unintended adopted less modern (all non-traditional) contraceptive methods overall, but adopted long-acting reversible contraception (LARC) more frequently than women reporting an intended pregnancy (OR 1.41; CI 1.18, 1.68). Among HIV-positive women, this relationship was particularly strong (OR 3.12; CI 1.96, 4.97). However, when examining changes in contraceptive use from the pre-pregnancy to the postpartum period, women who had an unintended pregnancy had lower odds of changing to a more effective method postpartum overall (OR 0.71; CI 0.64, 0.79).

**Conclusions:**

We did not find evidence of higher modern method adoption in the postpartum period among women with an unintended pregnancy. However, women who were already on a method in the pre-pregnancy period were catalyzed to move to more effective methods (such as LARC) postpartum. This study provides evidence of low modern (non-traditional) method adoption in general in the postpartum period among a vulnerable sub-population in Zimbabwe (women who experience unintended pregnancy). Simultaneously, however, it shows a relatively greater portion specifically of LARC use among women with an unintended pregnancy. Further research is needed to more closely examine the motivations behind these contraceptive decisions in order to better inform distribution and counseling programs.

**Electronic supplementary material:**

The online version of this article (10.1186/s12905-018-0668-z) contains supplementary material, which is available to authorized users.

## Background

Unintended pregnancy is associated with a spectrum of deleterious outcomes for women and their infants, including diminished use of prenatal health services [1, 2], increased maternal morbidity and mortality, unsafe abortion, and among young women, diminished economic prospects [3]. In addition, provision of family planning is an effective and cost-efficient strategy to ensure the reproductive rights of HIV infected women and to prevent vertical transmission of HIV at the population level by preventing unintended or mistimed pregnancies. Modern method uptake is thus a central objective of prevention of mother-to-child HIV transmission (PMTCT) strategies worldwide [4–6]. However, in 2014, 58% of women of reproductive age in sub-Saharan Africa who did not want to become pregnant were not using modern contraception [7]. As a result, increasing women’s access to and demand for family planning, including long-acting reversible contraception (LARC) methods that do not rely on daily adherence, is a global priority [8–11], particularly as it effectively improves women’s health through child spacing [12]. There has been much progress toward this goal, though LARC is still not universally available in sub-Saharan Africa. When LARC is available, a lack of knowledge and understanding of the method can be a barrier to use. The antenatal and postpartum periods, where women have increased contact with the health system, have been recognized as an optimal time for intervention with contraceptive and other health care messages [13]. The antenatal period may also be particularly important for HIV-positive women, as some studies show that they experience a greater burden of unintended pregnancy and may be interested in using modern contraception after delivery [14, 15]. Current evidence from sub-Saharan Africa on modern method use in the postpartum period suggests that there has been improvement in adoption over the years, and overall rates of use remain moderate [16–18].

Here we use data from a community-based survey of postpartum women in Zimbabwe to explore the relationship between pregnancy intention and contraceptive use in the postpartum period. We do this through three key analyses that examine: 1) the relationship between pregnancy intention and postpartum method choice, 2) differences in this relationship by HIV status, and 3) changes in contraceptive use from the prenatal to the postpartum periods.

## Methods

### Study population and data collection

The present secondary data analysis utilizes 2014 data from a serial cross-sectional survey of mother-infant pairs living in five of Zimbabwe’s ten provinces, conducted as part of an impact evaluation of Zimbabwe’s Accelerated National PMTCT Program [19]. Eligible participants were all mothers or caregivers over 16 years of age with infants aged 9 to 18 months [20]. Women who were HIV-negative, living with HIV infection, or of unknown HIV status were eligible. The impact evaluation methodology has been previously described [19–21]. In brief, mothers or caregivers were selected through a two-stage process that randomly sampled 1) catchment areas of facilities that provided maternal, newborn and child health services, and then 2) households (identified through immunization records, community health workers, and participant referrals). In addition to individual-level data, the impact evaluation collected information from clinics serving sampled catchment areas, including contraceptive method availability.

In-person questionnaires were administered by surveyors in 2014 at participants’ residences in their preferred language. Survey questions included sections on demographic characteristics, household composition, healthcare utilization, delivery, family planning preferences, and health status. Follow-up surveys were administered in later years as part of the study, but are not included in the present analysis.

### Inclusion criteria

Participants from the survey were included in the current analysis if they were a biological mother (rather than caregiver), had information on both pregnancy intention and contraceptive use, were not pregnant at the time of the survey, and did not report permanent sterilization of their male partner or hysterectomy in the postpartum period (*n* = 31).

### Key variables of interest

**Fig. 1:**
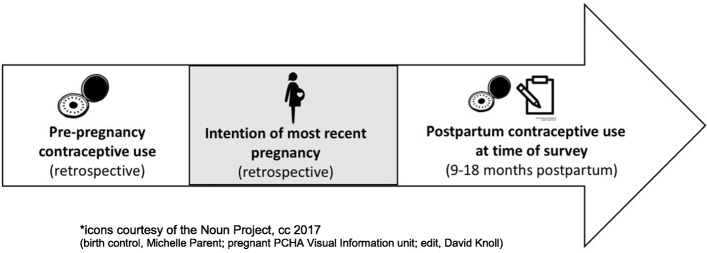
Pregnancy timeline among a sample of Zimbabwean women, surveyed at 9 to 18 months postpartum, 2014

The primary outcome was postpartum contraceptive use, defined as the method the woman reported using at the time of the survey (9 to 18 months postpartum; Fig. 1). The outcome was divided into three categories: 1) long-acting reversible contraception (LARC, highly effective methods that do not rely on adherence, including IUD and implants), 2) other modern methods (injectables, pill, condoms or diaphragm), or 3) no method or traditional method (referent group). Women who used dual methods were categorized into the group representing the more effective method, according to the Center for Disease Control’s taxonomy of method effectiveness [22].

To examine changes in contraceptive method from the pre-pregnancy to the postpartum period, we used the CDC’s effectiveness tiers [22] which included a ‘highly effective’ tier (LARC), an ‘effective’ tier (injectables, pill, patch), a ‘moderately effective’ tier (condoms, sponge, traditional methods), and ‘no method’ [22]. Method change was assessed by whether a woman in the postpartum period adopted: 1) a more effective method than her reported use pre-pregnancy, 2) a less effective method, or 3) stayed within the same method tier in the postpartum period (referent group).

Pregnancy intention was measured using an adaptation of the London Measure of Unplanned Pregnancy [23], which asked women whether their pregnancy came earlier than expected (mistimed), later than expected, when expected, or was not desired at all. Consistent with prior literature [1], women who had their pregnancy at the time desired or later than expected were combined into one “intended” category; women with earlier than desired or unwanted pregnancies were combined into a single group, labeled “unintended pregnancy”.

HIV status was self-reported based on a composite measure derived from reports of HIV status before or during the most recent pregnancy, and at labor and delivery but not at the time of the survey to ensure that self-report HIV status temporally preceded the outcome (postpartum contraceptive use).

### Statistical analysis

#### Objective 1

To examine the relationship between pregnancy intention and postpartum contraceptive use, we constructed a multinomial logistic regression model with unintended pregnancy as the independent variable and postpartum contraceptive use as a 3-level outcome—LARC, other modern method, or no method/traditional method (reference group).

#### Objective 2

We tested for effect modification by reported HIV status by adding an interaction term to the unadjusted model described above between pregnancy intention and postpartum use, excluding women with unknown HIV status (*n* = 370). The *p*-value on the interaction term was used to evaluate homogeneity between categories of HIV status; a *p*-value of less than 0.05 was considered evidence of effect modification.

#### Objective 3

To examine changes in contraceptive method before and after the pregnancy, a separate multinomial logistic regression model was constructed using the 3-level dependent variable ‘method change’, described above.

Analyses were conducted using STATA version 13.0 (College Station, Texas). Observations were weighted to account for varying sampling fractions across catchment areas.

### Covariate selection

Based on previous literature [8–10, 24], we considered potential confounding by education, age, marital status, parity, ethnicity, assets, and province. We measured assets as a proxy for income, using a principal components analysis of a household asset index (ownership of phone, television, etc.), similar to a previous analysis [20]. Additionally, HIV status, previous contraceptive method use, antenatal care (ANC) visits, and LARC availability at the catchment area clinic were considered as potential confounders. All potential covariates were considered for inclusion through analysis of a directed acyclic graph (DAG) [25]. Less than 1 % of any covariate was missing.

### Sensitivity analyses and mitigation of bias

Three sensitivity analyses were performed for the first objective. The first examined differences in the effect of pregnancy intention on subsequent postpartum contraceptive use by months postpartum (9–12 vs. 13–18 months), since it is possible that a woman’s recollection of pregnancy desires and contraceptive choices could change as she enters the more extended postpartum period. The second sensitivity analysis examined differences in the association among women who were or were not breastfeeding at the time of the survey, as breastfeeding women may rely more heavily on natural methods of avoiding pregnancy given lactational amenorrhea. The third sensitivity analysis examined differences in the association according to whether or not women lived in the catchment area of a clinic that reported having LARC available (since, despite increased coverage from country-wide distribution campaigns, not all clinics in the sample reported offering LARC). This was self-reported by the in-charge of the facility, and was the closest proxy for provision available from our data.

For the third objective, a sensitivity analysis was performed stratifying results by months postpartum (9–12 vs. 13–18 months) and restricting to women who were not using a method in the pre-pregnancy period.

All sensitivity analyses were evaluated for a > 10% change in the main effect coefficient (pregnancy intention).

## Results

### Participant demographics and unintended pregnancy

**Fig. 2:**
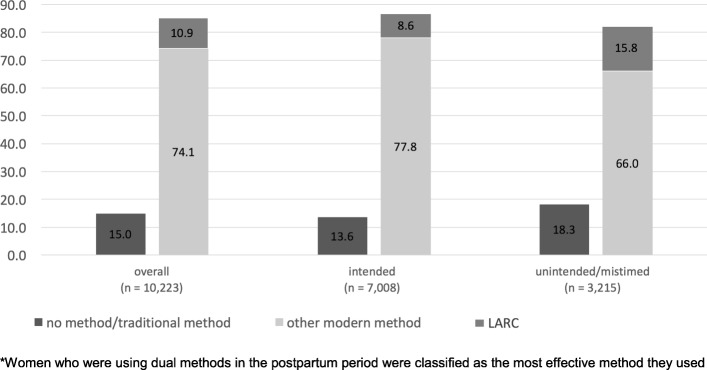
Percent of postpartum method use^a^ by intention exposure among women in Zimbabwe, 2014

Of the 10,646 participants in the 2014 survey, 10,058 (95%) met inclusion criteria and were included in the current analysis (weighted *n* = 10,223). The mean age was 27, and 80% of the women included in the sample were married and cohabitating with their spouses (Table 1). Overall uptake of modern contraception was high (85%, Fig. 2). In the sample, 3215 (31%) of the women reported that their pregnancy was unintended. Women who reported that their pregnancy was unintended were more likely to be unmarried, to be in the lowest income quartile, to report HIV-positive status, to have a lower educational status, to have access to LARC at the facility in their catchment area and to be using a contraceptive method in the pre-pregnancy period (Tables 1 and 2). Notably, in the pre-pregnancy period 36% of women with an intended pregnancy were on a method in the pre-pregnancy period (Table 2).

**Table 1:** Demographic characteristics of postpartum Zimbabwean women at 9–18 months postpartum residing in five provinces, overall and by pregnancy intention, 2014^a^

	Overall	Intended pregnancy	Unintended pregnancy	*p*-value^b^
	N (%)	N (%)	N (%)	
Total	10,224 (100)	7008 (100)	3215 (100)	
Age (mean, SE)	(26.8, 0.10)	(26.8, 0.11)	(26.6, 0.11)	0.08
Education				< 0.01*
No school	174 (1.7)	125 (1.8)	49 (1.5)	
Primary school	2301 (22.8)	1546 (22.1)	781 (24.3)	
Some secondary	2714 (26.9)	1819 (26.0)	934 (29.1)	
Grade “O” and above	4902 (48.6)	3516 (50.2)	1451 (45.1)	
Ethnicity				<.01*
Shona	8847 (87.7)	6285 (89.7)	2675 (83.2)	
Ndebele	611 (6.1)	333 (4.8)	291 (9.1)	
Kalanga	90 (0.9)	55 (0.8)	36 (1.1)	
Other	542 (5.4)	334 (4.8)	213 (6.6)	
Marital status				<.01*
Married and cohabitating	8108 (80.4)	5891 (84.1)	2311 (71.9)	
Married but not cohabitating	1179 (11.7)	799 (11.4)	402 (12.5)	
Divorced/separated/widowed	99 (1.0)	278 (4.0)	364 (11.3)	
Never married	175 (1.7)	40 (0.6)	138 (4.3)	
Parity (mean, SE)	(2.6, 0.05)	(2.5, 0.05)	(2.7, 0.05)	<.01*
Province				<.01*
Harare	3550 (35.2)	2438 (34.8)	1143 (35.6)	
Manicaland	2796 (27.7)	2059 (29.4)	776 (24.1)	
Mashonaland Central	1545 (15.3)	1096 (15.7)	472 (14.7)	
Mashonaland West	1389 (13.8)	958 (13.7)	451 (14.0)	
Matabeleland South	810 (8.0)	457 (6.5)	372 (11.6)	
Number of ANC visits (mean, SE)	(4.8, 0.08)	(4.9, 0.07)	(4.4, 0.09)	<.01*
Asset index				0.27
1st quartile	2125 (20.8)	1417 (20.2)	708 (22.0)	
2nd quartile	1988 (19.4)	1372 (19.6)	616 (19.1)	
3rd quartile	2289 (22.4)	1563 (22.3)	727 (22.6)	
4th quartile	3821 (37.4)	2656 (37.9)	1165 (36.2)	
HIV status				<.01*
Positive	958 (9.4)	545 (7.8)	448 (13.9)	
Negative or unknown	9265 (90.6)	6463 (91.7)	2767 (86.4)	
LARC available at the local clinic	6173 (60.4)	4170 (59.5)	2003 (62.3)	0.05*

*Significant at the *p* = 0.05 level

^a^Data weighted to account for varying sampling fraction across catchment areas

^b^Continuous data assessed using t-tests; categorical data assessed using chi-squared tests

**Table 2:** Pre-pregnancy and postpartum contraceptive use, stratified by pregnancy intention, among postpartum women in Zimbabwe, 2014

	Pre-pregnancy				9–18 months Postpartum			
	Intended		Unintended		Intended		Unintended	
	N	%	N	%	N	%	N	%
No method/ natural method	4488	64.1	1456	45.3	950	13.6	587	18.3
Other modern method	2445	34.9	1734	53.9	5454	77.8	2120	66.0
LARC (IUD, implant)	74	1.1	25	0.8	604	8.6	508	15.8
Total	7008	100.0	3215	100.0	7008	100.0	3215	100.0

### Association between pregnancy intention and postpartum contraceptive method

In the postpartum period, 11% of the women in the sample reported LARC use, 74% reported other modern method use, and 15% reported no method or traditional method use (Table 2). Among women who had an unintended pregnancy, 16% reported LARC use postpartum, compared to 9% of those with an intended pregnancy (Fig. 2, Table 3). Women with an unintended pregnancy were less likely to use other modern methods (66% vs. 78%; Fig. 2, Table 3), and more likely to use traditional or no contraceptive methods (18% vs. 14%; Fig. 2, Table 3) than women with intended pregnancies. After adjustment for covariates, compared to women with intended pregnancies, women who reported that their pregnancy was unintended had 1.41 times the odds of adopting LARC (95% CI 1.18, 1.68; Table 3) and 0.80 times the odds of adopting other modern methods in the postpartum period (95% CI 0.70, 0.92; Table 3). Results were similar, though less precise, in sensitivity analyses (Additional file 1: Appendix A).

**Table 3:** The association between pregnancy intention and postpartum contraceptive method use, overall and stratified by self-reported HIV status, among women with a recent birth in Zimbabwe, 2014^f^

Pregnancy intention	LARC^a^ Use (referent: no method/ natural method)				Modern Method Use^b^ (referent: no method/ natural method)				No method/natural method (referent)	
	N	%^c^	Crude OR^d^ (95% CI^d^)	Adj^e^ OR (95% CI)	N	%	Crude OR (95% CI)	Adj. OR (95% CI)	N	%
Overall										
Unintended	508	15.8	1.36 (1.10, 1.69)	1.41 (1.18, 1.68)	2120	66.0	0.63 (0.53, 0.74)	0.80 (0.70, 0.90)	587	18.3
Intended (Referent)	604	8.6	–	–	5454	77.8	–	–	950	13.6
HIV positive										
Unintended	102	22.8	2.57 (1.64, 4.02)	3.12 (1.96, 4.97)	290	64.7	1.09 (0.72, 1.65)	1.52 (1.01, 2.30)	56	12.6
Intended (Referent)	60	11.0	–	–	400	73.4	–	–	85	15.6
HIV negative										
Unintended	406	14.7	1.19 (0.96, 1.49)	1.30 (1.08, 1.58)	1831	66.2	0.58 (0.49, 0.69)	0.75 (0.65, 0.88)	531	19.2
Intended (Referent)	544	8.4	–	–	5055	78.2	–	–	865	13.4

^a^Long Acting Reversible Contraception(LARC) includes intrauterine device (IUD) and the implant

^b^Modern methods include the pill, injectables, condoms and diaphragm

^c^Percent by stratum (overall, HIV positive, HIV negative respectively)

^d^*OR* odds ratio, *CI* confidence interval

^e^Adjusted for ethnicity, age, asset quartile, marital status, parity, HIV status, education, number of ANC visits and pre-pregnancy contraceptive use

^f^Interaction terms for both levels of the multinomial (LARC and other modern method use) were significant at the <.01 level

### Effect modification by HIV status

We found evidence of effect modification by HIV status. Among women who identified themselves as HIV-positive (prior/during pregnancy or during delivery), those reporting an unintended pregnancy had 3.12 times the adjusted odds of postpartum LARC use, compared to HIV-positive women reporting an intended pregnancy (95% CI 1.96, 4.97; Table 3). While the direction of the effect of pregnancy intention on postpartum LARC use held among HIV-negative women, the effect was nonsignificant (adjusted OR 1.19; CI 0.96, 1.49; Table 3). HIV-positive women with an unintended pregnancy also had nonsignificant increased odds of postpartum other modern method use compared to HIV-positive women with an intended pregnancy (OR 1.09; CI 0.72, 1.65).

### Unintended pregnancy and method change

Overall, 54% of women adopted a more effective method in the postpartum period, 42% adopted a method of similar effectiveness, and 5% adopted a less effective method. Those who experienced an unintended pregnancy were less likely to adopt a more effective method in the postpartum period relative to their pre-pregnancy use, compared to women reporting an intended pregnancy (adjusted OR 0.71, 95% CI 0.64, 0.79). Women reporting an intended pregnancy were more likely to be using no method (tier 1) prior to their most recent pregnancy (64%, compared to 45% of women who reported that their pregnancy was unintended, Table 2)—likely due to trying to become pregnant—and therefore had more opportunity to move to a more effective tier. Although overall women with an unintended pregnancy were more likely to switch to less effective methods, greater portions of women who reported that their pregnancy was unintended switched to highly effective (tier 4) methods in the postpartum period, regardless of method effectiveness use pre-pregnancy (Additional file 1: Appendix B). There was no significant difference in the effect of pregnancy intention on method change by month postpartum (9–12 vs. 13–18 months, data not shown). The direction of the effect was the same also when examined only among women on no method in the pre-pregnancy period (data not shown).

## Discussion

We examined the relationship between pregnancy intention and postpartum contraceptive use among a representative sample of women 9–18 months postpartum from five provinces in Zimbabwe. We found that women reporting that their pregnancy was unintended were less likely to adopt some types of modern contraception (pills, injectables, condoms and diaphragm) in the postpartum period. However, they had greater odds of LARC uptake in the postpartum period. The relationship between pregnancy intention and LARC use was present irrespective of the number of months postpartum or breastfeeding status, and is strongest among women living with HIV. In examining uptake of effective method over time, we found that over half of women in the sample adopted more effective methods in the postpartum period. However, although women who reported that their pregnancy was unintended were more likely to use LARC post-partum, overall they were more likely to switch to a less effective method between the pre-pregnancy and postpartum periods. These data suggest that women with an unintended pregnancy are not more motivated than others to adopt modern methods or more effective methods in general in the postpartum period in order to prevent future pregnancy. Notwithstanding, women experiencing an unintended pregnancy are catalyzed to adopt LARC (highly effective) methods. LARC methods are the most effective reversible methods on the market, and do not rely on daily or quarterly adherence. These “set and forget” methods may be particularly attractive to women who have experienced an unintended pregnancy. This effect be particularly true among a subpopulation of women who were already on an effective method (pill, patch, etc.) pre-pregnancy, and are therefore more economically, socially, personally and/or physically prepared to adopt LARC.

Historically, provision of LARC in Zimbabwe has been limited [26]. However, the Ministry of Health and Child Care, through a range of partners, were recently funded to increase the supply of LARC throughout the country as a population-level family planning strategy. We conducted a sensitivity analysis among facilities reporting LARC provision, however, given the rapid expansion of LARC from various implementing partners in the country, this may be an imperfect of LARC supply. Notwithstanding, the increased odds of postpartum LARC use among women who report unintended pregnancy—particularly among HIV-positive women—is a mark of demand in a key sub-population of HIV-positive women who had experienced unintended pregnancy. The postpartum period in particular is a key time for contraceptive choice in order to ensure maternal and infant health through prevention of unintended pregnancy and lengthened child spacing. Women may also be more receptive to behavior change in the postpartum period; it is a sensitive period in which women redefine their identity as new or continuing mothers.

While adequate provision of highly effective methods would lessen the burden of unintended pregnancy, LARC is far from a panacea. Many factors influence method choice, including stigma and misperceptions, side effects, relationship context, and other personal and social reasons [27, 28]. Given that women who reported unintended pregnancy adopted less modern methods overall, these and other reasons must be more closely examined in order to delineate the motivations behind the lower modern method adoption. It is possible, for example, that women who experience an unintended pregnancy are dissatisfied with their pre-pregnancy method choice, but due to misinformation or personal choice, adopt a less effective method (or none at all) postpartum.

The study has several strengths, including a large and representative sample and community-based design. To minimize social desirability bias, the survey was conducted anonymously. The analysis is limited, however, by the self-reported and retrospective nature of the exposure of interest, pregnancy intention. Women with HIV in particular may feel pressured to over-report unintended pregnancy and family planning use, due to pressure from providers or the community not to conceive. In this survey, HIV status is also self-reported, and women may underreport their status. While the team recognizes this may introduce bias in the analysis, the analysis seeks to quantify the relationship between how a woman’s knowledge of her own status may interact with pregnancy intention; therefore self-report is still an appropriate measure.

## Conclusions

The present study demonstrates that Zimbabwean women who report that their pregnancy is unintended have lower odds of modern method use overall, but greater odds of adopting LARC postpartum. It supports literature that finds a negative association between intention and postpartum modern method use contraceptive use [8], but adds the unique finding of a positive association for LARC, specifically in the Zimbabwean context. We suggest further research to better understand the factors—both personal and social—that contribute to postpartum contraceptive decisions for women with an unintended pregnancy, particularly in Zimbabwe. A more nuanced understanding of these motivations relative to pregnancy intention would lead to programs more sensitive to the needs, intentions, and desires of women, and therefore more prepared to support them through informed contraceptive decisions in the postpartum period.

## Additional file

Additional file 1: **Appendix A.** The association between pregnancy intention and postpartum contraceptive method use, stratified by months postpartum and breastfeeding status, among women with a recent birth in Zimbabwe, 2014. **Appendix B.** Flowchart of effectiveness of method choice, from pre-pregnancy to postpartum periods, stratified by pregnancy intention among women in Zimbabwe, 9-18 months postpartum. (ZIP 216 kb)
