# Supplementary material for: Unintended pregnancy and subsequent postpartum long-acting reversible contraceptive use in Zimbabwe
Source: BMC Womens Health. 2018 Nov 26;18:193. doi: 10.1186/s12905-018-0668-z (PMC6258256; doi:10.1186/s12905-018-0668-z)
Supplement: Supplementary file 1 — Appendix A. The association between pregnancy intention and postpartum contraceptive method use, stratified by months postpartum and breastfeeding status, among women with a recent birth in Zimbabwe, 2014. Appendix B. Flowchart of effectiveness of method choice, from pre-pregnancy to postpartum periods, stratified by pregnancy intention among women in Zimbabwe, 9-18 months postpartum. (ZIP 216 kb) [file 12905_2018_668_MOESM1_ESM.zip › Appendices_revisedR1.docx]

Appendix A: The association between pregnancy intention and postpartum contraceptive method use, stratified by months postpartum and breastfeeding status, among women with a recent birth in Zimbabwe, 2014

|  | | LARC^a^ Use  *(referent: no method/* *natural method)* | | | Modern Method Use^b^  *(referent: no method/* *natural method)* | | |
| --- | --- | --- | --- | --- | --- | --- | --- |
| Population | Pregnancy intention | **N** | **%** | **Adj^c,d,e^ OR (95% CI)** | **N** | **%** | **Adj. OR**  **(95% CI)** |
| **Women 9-18 months postpartum**  **(full sample)** | **Unintended** | 508 | 15.8 | 1.44  (1.20, 1.72) | 2120 | 66.0 | 0.80  (0.70, 0.90) |
|  | **Intended (Referent)** | 604 | 8.6 | — | 5454 | 77.8 | — |
| **Women 9-12 months postpartum^f^** | **Unintended** | 204 | 14.3 | 1.52  (1.15, 2.00) | 990 | 69.3 | 0.92  (0.77, 1.11) |
|  | **Intended (Referent)** | 247 | 8.0 | — | 2421 | 78.3 | — |
| **Women 13-18 months postpartum^f^** | **Unintended** | 304 | 17.0 | 1.34  (1.02, 1.73) | 1128 | 63.3 | 0.72  (0.61, 0.85) |
|  | **Intended (Referent)** | 357 | 9.1 | — | 3025 | 77.5 | — |
| **Breastfeeding women** | **Unintended** | 403 | 14.8 | 1.36  (1.10, 1.68) | 1837 | 67.4 | 0.77  (0.68, 0.87) |
|  | **Intended (Referent)** | 488 | 8.1 | — | 4790 | 79.1 | — |
| **Non-breastfeeding women** | **Unintended** | 97 | 21.2 | 1.53  (0.90, 2.61) | 265 | 57.8 | 0.93  (0.63, 1.38) |
|  | **Intended (Referent)** | 107 | 12.3 | — | 611 | 70.1 | — |
| **LARC available in catchment** | **Unintended** | 345 | 17.2 | 1.44  (1.15, 1.78) | 1297 | 64.7 | 0.86  (0.70, 1.05) |
|  | **Intended (Referent)** | 421 | 10.1 | — | 3180 | 76.3 | — |
| **LARC unavailable in catchment** | **Unintended** | 426 | 15.9 | 1.45  (1.17, 1.81) | 1770 | 65.8 | 0.78  (0.67, 0.90) |
|  | **Intended (Referent)** | 486 | 8.2 | — | 4634 | 78.5 | — |

1. Long Acting Reversible Contraception(LARC) includes intrauterine device (IUD) and the implant
2. Modern methods include the pill, injectables, condoms and diaphragm
3. Calculated for women 9-12 months postpartum (n = 4442), and women 13-18 months postpartum (n = 5598), additional exclusions were made in this analysis due to missing child age variable (n=18 exclusions); also calculated for women breastfeeding (n=8784) and not breastfeeding (n=1329), additional exclusions were made for those missing the breastfeeding variable (n=110)
4. OR=odds ratio; CI= confidence interval
5. Adjusted for ethnicity, age, asset quartile, marital status, parity, HIV status, education, pre-pregnancy contraceptive use and number of ANC visits
6. Interaction term between timing postpartum and contraceptive use was significant at the p=0.05 level for other modern method use (p=0.01), but not for LARC use (p=0.40)

Appendix B: Flowchart of effectiveness of method choice, from pre-pregnancy to postpartum periods, stratified by pregnancy intention among women in Zimbabwe, 9-18 months postpartum

a) All counts presented are weighted by district size

b) Contraceptive categories created using definitions from the CDC infographic 'Effectiveness of Family Planning Methods' (Centers for Disease Control and Prevention, 2011)
c) Tier 4: highly effective methods, resulting less than one birth per 100 women per year, includes long-acting reversible contraception (IUD and implant)
d) Tier 3: effective methods, resulting in 6-12 pregnancies per 100 women per year, includes injectables, pill, patch, ring, or diaphragm 
e) Tier 2: moderately effective methods, resulting in 18 or more pregnancies per year, includes male and female condoms, withdrawal, sponge, spermicide and fertility-based awareness
f) Tier 1: no method used
