# Supplementary figures and images for: Unintended pregnancy and subsequent postpartum long-acting reversible contraceptive use in Zimbabwe
Source: BMC Womens Health. 2018 Nov 26;18:193. doi: 10.1186/s12905-018-0668-z (PMC6258256; doi:10.1186/s12905-018-0668-z)

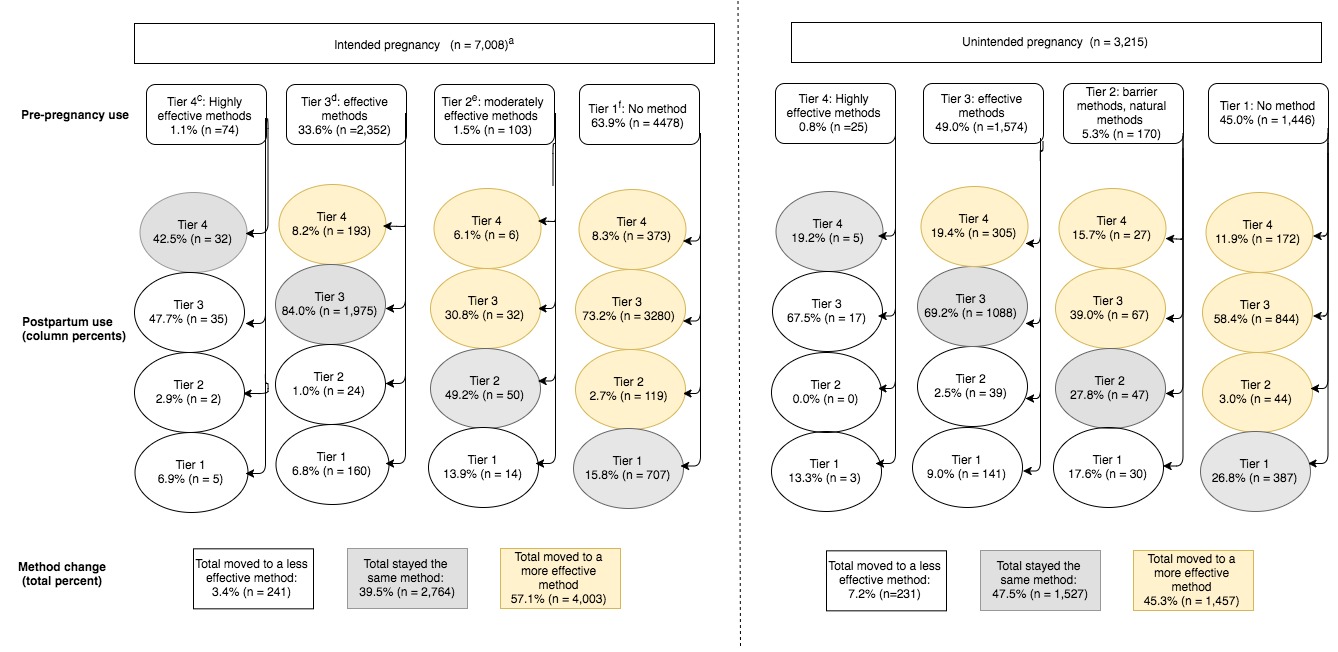

Supplement: Supplementary file 1 — Appendix A. The association between pregnancy intention and postpartum contraceptive method use, stratified by months postpartum and breastfeeding status, among women with a recent birth in Zimbabwe, 2014. Appendix B. Flowchart of effectiveness of method choice, from pre-pregnancy to postpartum periods, stratified by pregnancy intention among women in Zimbabwe, 9-18 months postpartum. (ZIP 216 kb) [file 12905_2018_668_MOESM1_ESM.zip › Fig BR1.jpg]
